# Mobile health-based home rehabilitation education improving early outcomes after anterior cruciate ligament reconstruction: A randomized controlled clinical trial

**DOI:** 10.3389/fpubh.2022.1042167

**Published:** 2023-01-12

**Authors:** Yi Guo, Dai Li, Yi-bo Wu, Xin Sun, Xin-ying Sun, Yu-ping Yang

**Affiliations:** ^1^Beijing Key Laboratory of Sports Medicine and Joint Injuries, Department of Sports Medicine, Peking University Third Hospital, Peking University Institute of Sports Medicine, Beijing, China; ^2^School of Public Health, Peking University Health Science Center, Beijing, China; ^3^Department of Sports Medicine, Peking University Third Hospital, Beijing, China

**Keywords:** anterior cruciate ligament reconstruction, health education, knee function, mobile health (mHealth), health belief model, structural equation model (SEM)

## Abstract

**Objective:**

This study aimed to assess changes in joint range of motion (ROM) and knee joint function between patients who received the mobile health-based intervention and those who received regular care at 2 and 6 weeks after anterior cruciate ligament (ACL) reconstruction to provide better interventions in the future.

**Methods:**

Patients who underwent ACL reconstruction were randomized into the experimental [Mobile health-based intervention (MHI); *n* = 62] and control (CON) groups (*n* = 63). The CON group underwent home-based rehabilitation exercise following the paper rehabilitation schedule, while the intervention group received additional mobile health-based education at weeks 1–6 after surgery. ROM, thigh circumference difference, and flexion pain were the primary outcomes. The secondary outcomes were the international knee documentation committee knee evaluation form (IKDC) scores and rehabilitation compliance scores. All the outcomes were measured 1 day before surgery as references and at 2 and 6 weeks after surgery.

**Results:**

There was no statistical difference in the patients' ROM, thigh circumference difference, and VAS scores at the 2-week follow-up. At the 6-week follow-up, the ROM of the affected leg was (118.1 ± 20.5)° in the CON group and (126.6 ± 20.5)° in the MHI group, and the difference was statistically significant (*P* = 0.011). The difference in thigh circumference was 3.0 (2.0, 3.5) cm in the CON group and 2.5 (1.0, 3.0) cm in the MHI group. The difference was statistically significant (*P* < 0.001). The VAS score in the CON group was 3.0 (2.0, 4.0), and the MHI group was 2.5 (1.0, 3.0). The difference was statistically significant (*P* < 0.05). At the 6-week follow-up, the compliance score of patients in the MHI group was significantly higher than that in the CON group (*P* = 0.047, β = 2.243, 95%CI: 0.026–4.459). There is no statistically significant difference in IKDC scores.

**Conclusion:**

Mobile health-based intervention positively affected patients undergoing ACL reconstruction surgery, particularly in improving the clinical outcome indicators of the knee joint.

## What does this study add to existing knowledge?

- The rehabilitation management of patients after ACL reconstruction can be implemented according to the Health belief model.
- Mobile health-based intervention can improve patients' knee function, muscle atrophy, and joint pain by 6 weeks after ACL reconstruction.
- Mobile health-based education further promoted the improvement of clinical indicators but did not significantly improve the subjective results after ACL reconstruction.

## Introduction

With the increase in people's health awareness, more residents are engaged in physical exercise, which has led to annual increases in the incidence of anterior cruciate ligament (ACL) injuries (1, 2). ACL injuries account for about 40% of knee sports injuries (3). ACL reconstruction has been proven to be the best treatment for ACL injuries (3). Postoperative rehabilitation requires frequent physiotherapy sessions for both therapy and education (4). To ensure the functional rehabilitation of the knee joint after the operation, make the patient reach the rehabilitation goal according to the plan, and reduce the risk of the second operation, it is essential to guide the patient to carry out the proper rehabilitation training (5). However, owing to limited rehabilitation medical resources (6, 7), most patients lack professional rehabilitation guidance after undergoing ACL reconstruction after discharge. Most patients can only refer to the paper rehabilitation schedule prescribed by the rehabilitation physician after release. This results in poor compliance, making the target results challenging and affecting the patient's quality of life (8).

Mobile health (mHealth) facilities (smartphone-based educational apps, web-based tools, SMS text messaging, PDA physiological status monitoring, and connected captors) have been widely proven to be effective interventions in different health areas, which can realize the transition from “one-to-one” to “one-to-many” for doctors and patients (9, 10), making management more time-effective. And with the development of Internet technology, personalized management of patients has gradually become possible. MHealth-based education has been widely used in different health fields, such as managing behaviors in patients with chronic diseases (11, 12) and improving patient compliance after discharge (13, 14). Mhealth has also been applied to the out-of-hospital rehabilitation management of patients with sports injuries (15). Therefore, mHealth rehabilitation intervention may be more effective than traditional care. Mhealth intervention also provides new ideas to ensure rehabilitation in outpatients under the normalized management of coronavirus disease, reducing the risk of cross-infection in hospitals. Limited by evidence, whether mHealth-based education can reduce the burden of rehabilitation physicians and effectively improve the process and results of patient rehabilitation remains to be elucidated.

Regarding intervention content, the Health belief model (HBM) is one of the most widely used theories to examine the barriers and foundation of individual participation in programs that focus on preventing diseases and promoting healthy lifestyles (16). HBM enables the prediction of behaviors according to constructs consisting of perceived susceptibility, perceived severity, perceived benefits, perceived barriers, cues to action, and self-efficacy (17) and has been proven efficient in standardizing the rehabilitation exercise after hemodialysis (18), assisting patients with knee joint fractures to recover at home after surgery (17). Therefore, education strategies that the health belief model guides may be more effective than conventional interventions.

Therefore, the researchers have developed a mHealth-based intervention applet [MHI; “Rehabilitation Cloud Platform” (Beijing QianriQianyue Technology Ltd.)] for patients. The intervention content of the applet is designed based on HBM. This randomized controlled study aimed to compare the changes in knee joint clinical and subjective functional indicators in the MHI and the CON groups at 2 and 6 weeks after ACL reconstruction. This study hypothesized that a mobile health intervention would be more beneficial to the early recovery of patients after ACL reconstruction.

## Methods

### Study design and participants

This is a randomized controlled trial in which patients were tested 1 day before surgery and 2 and 6 weeks after surgery. The patients were individually randomized to one of two parallel groups in a 1:1 ratio to receive either conventional or conventional care plus mHealth-based intervention. Physiotherapists divided the patients into two parallel groups according to the grouping tool designed by the statistician, who was not involved in the data collection. Group allocation was completed before surgery. Except for the physiotherapists administering the treatment, the patients and data collectors were blinded to the patients' group assignments.

Patients awaiting ACL reconstruction were recruited at Peking university third hospital from April 2019 to December 2019 under the direction of five orthopedic surgeons. The inclusion criteria were as follows: (1) age between 18 and 60 years; (2) isolated ACL reconstruction for the first time, which can be combined with cartilage injury and partial meniscus resection; (3) a consistent postoperative recovery plan; (4) essential reading and writing skills and no communication problems; (5) ability to use smartphones with WeChat installed. The patient exclusion criteria were as follows: (1) the previous history of joint infection, joint tuberculosis or osteomyelitis, or lower limb surgery within 6 months; (2) severe heart, brain, kidney, and other organ dysfunctions; (3) combined with other severe knee joint diseases and injuries; (4) patients with mental illness or cognitive impairment; (5) transfer to other medical institutions after discharge.

The pre- and post-injury physical activity levels were determined using the ROM.

### Surgical technique

All the ACL reconstructions for patients enrolled in the study were performed *via* anteromedial portal with autogenously hamstring single-bundle reconstruction. The femoral side of the autograft was fixated with Endobutton (Smith and Nephew, USA), and the tibial side was fixated to the tibial tunnel with Intrafifix (Smith and Nephew, USA).

### Intervention procedures

In the CON group, patients were given a paper version of the rehabilitation plan before they were discharged from the hospital. A brief explanation was given to explain the plan and answer any related questions from the patients. The length of the explanation depended on the patient's level of understanding and was usually 5–10 min. After discharge from the hospital, the patients conducted rehabilitation training according to the plan, without any visits to other institutions or inpatient treatment. The control group could only use the data measurement function in the “Rehabilitation Cloud Platform” and could not use the rehabilitation instruction function, thus avoiding contamination in the control group.

In the MHI group, the mHealth intervention included these contents: sports medicine doctors will teach the patient how to properly use the “Rehabilitation Cloud Platform” the day before their surgery. The teaching included measuring data such as angle and circumference and how to view the rehabilitation guidance content. The platform then informs the patient before surgery about the knowledge related to the surgery and the importance of rehabilitation. In the home-based rehabilitation stage, the platform will remind the patient to complete their rehabilitation plan and explain the details of the plan for their reference. Patients can upload relevant data according to their needs and the platform's suggestions. The platform can provide feedback instructions to patients according to their uploaded data and suggest whether they should seek medical treatment in time.

**Figure 1:**
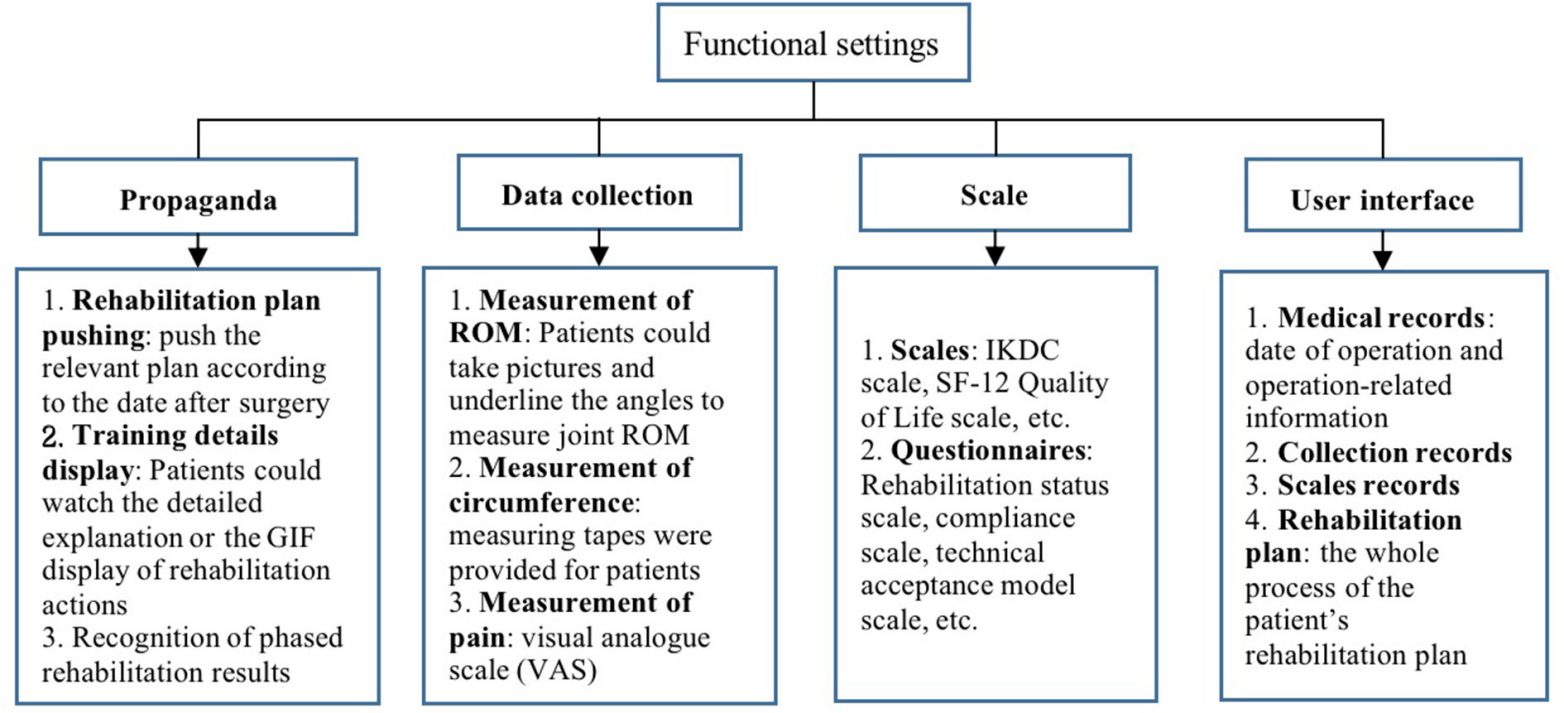
Function settings of the “Rhabilitation Cloud Platform.” The figure shows the four functions of the “Rhabilitation Cloud Platform” and their detailed introduction.

**Figure 2:**
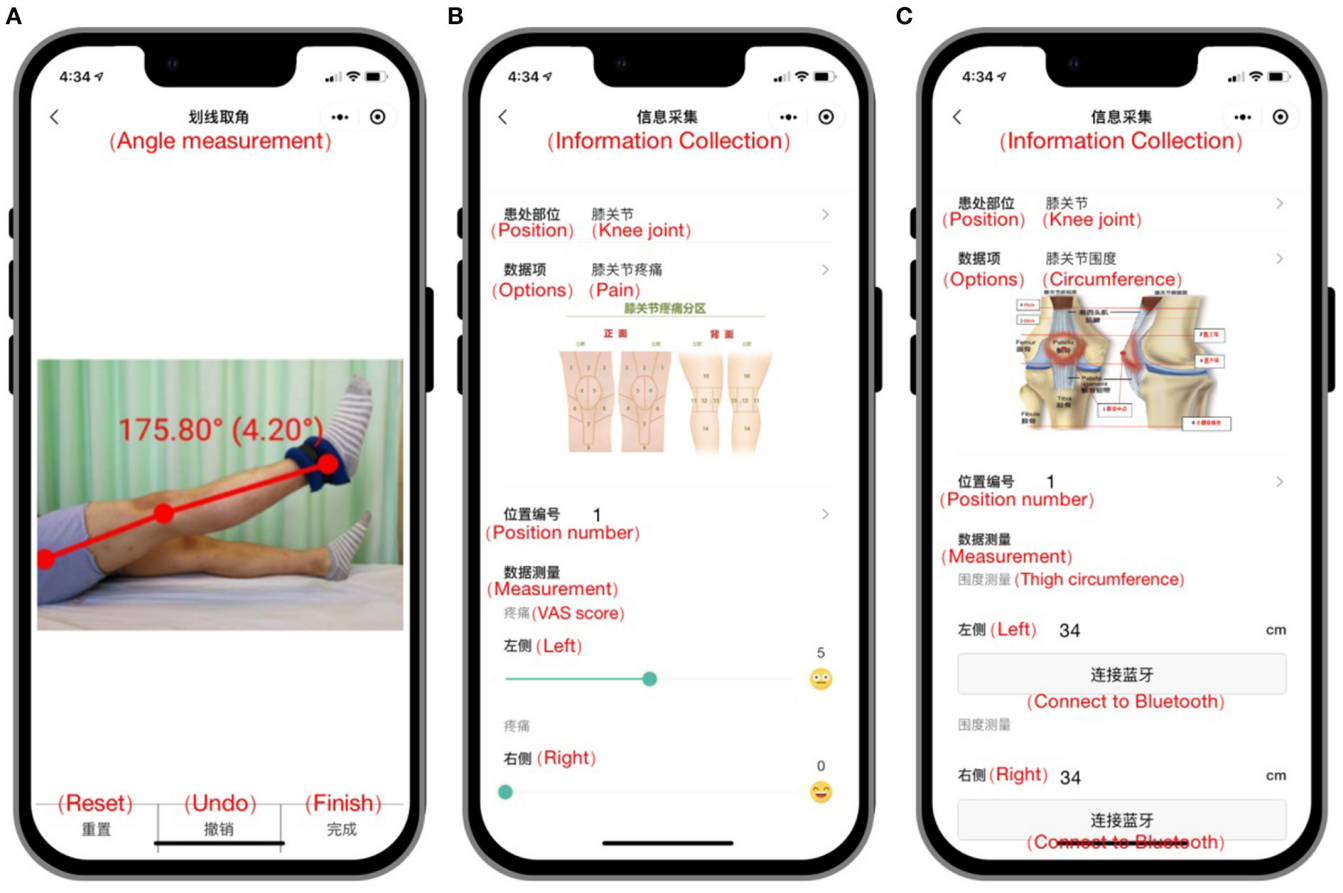
Data collection interface of the “Rhabilitation Cloud Platform.” **(A)** After the patient uploads the photo, the platform can automatically measure the ROM; **(B)** Patients can record the VAS score of knee flexion pain through the platform; **(C)** Patients can record the thigh circumference through the platform.

In addition, the contact information of the doctor is also provided on the platform. If patients have questions about the teaching content in the platform, they can ask the doctor through the platform. The doctor would review and respond timely. The settings and content of the “Rehabilitation Cloud Platform” are shown in Figure 1 and Table 1, and the data collection system is shown in Figure 2.

**Table 1:** Rehabilitation exercise education after ACL reconstruction.

**Phase**	**Days**	**Content**	**HBM intervention**
Phase 1: mobilization	Surgery ±1	Focus on the propaganda of the importance of rehabilitation exercise and enhancing confidence in the recovery, including the following: information on the principles and procedures of ACL reconstruction; information on the causes and treatments of postoperative symptoms; and presentation of cases with complete postoperative recovery and the consequences of irregular postoperative rehabilitation exercise.	Self-efficacy, perceived severity, and perceived benefits
Phase 2: basic exercise	1–4	Standardization in the form of graphic interchange format and captions Propaganda of how to treat the pain after surgery correctly Information on risk signals that suggest medical attention in a timely manner Standardization of the ankle pump practice, muscle function exercise (quadriceps contraction and straight-leg lift), and ROM exercise (passive stretch)	Self-efficacy, perceived susceptibility, perceived barriers, and cues to action
Phase 3: advanced exercise	4–21	Standardization and compliance retention, in addition to previous exercises and precautions, and notifications, with the following exercises added: ROM exercise (passive bend) Muscle function exercise (side-lying leg lift) Daily notification on the training content and date of follow-up	Self-efficacy, perceived susceptibility, perceived barriers, and cues to action
Phase 4: maximum strength	21–42	Compliance retention Daily notification on the training content and date of the second follow-up Information on when the patient can resume normal activities by specific cases	Self-efficacy, perceived susceptibility, perceived barriers, and cues to action

### Outcome measures

#### Primary outcomes

The primary outcomes were ROM, the difference in thigh circumference, and the visual analog scale (VAS) score. Knee ROM (degrees) was assessed using the goniometer tool of the “Rehabilitation Cloud Platform,” with the participant lying supine following the previous procedures (19). ROM was measured before the operation and at 2 and 6 weeks postoperatively. The subjects were allowed a 3-min warm-up, which consisted of self-stretching within their available ROM. Knee extension measurements were taken with a towel roll under the heel of the involved extremity. Goniometric results measured with smartphone-based digital photography have been proven efficient in assessing joint function post-surgery (20, 21). The rehabilitation physician measured and recorded the difference in thigh circumference and the VAS score.

#### Secondary outcomes

The secondary outcomes were the IKDC score and rehabilitation compliance score. The online questionnaire on the “Rehabilitation Cloud Platform” was used for measurement.

The IKDC scale consists of 10 items on knee joint function and 8 items on knee ligament examination, including joint pain, exercise level, and daily activity ability, with a total score of 0–100. A higher score indicates better joint function. The IKDC scale is reliable, valid, and sensitive to changes in the joint functions of patients who underwent ACL reconstruction (22). The IKDC questionnaire is also always utilized in ACL research and is easier to complete and understand than other questionnaires (23, 24).

The rehabilitation compliance scale was compiled according to the content of the patient's rehabilitation schedule (2019 version) after ACL reconstruction in the sports medicine department of Peking university third hospital. According to the actual degree of completion of the patients' home rehabilitation training, a 5-point Likert scale was used for classification and scoring, with scores ranging from 1 (totally disagree) to 5 (totally agree) for each item, including ankle pump training, muscle contraction training, leg lifting, stretching and bending, ice regimen, and brace wearing.

### Sample size estimation and statistical analysis

ROM was used as the primary outcome, and the attainment value of ROM at 6 weeks was 120°, referring to the patient's rehabilitation schedule (2019 version) after ACL reconstruction in the sports medicine department of Peking university third hospital. Previous studies have mostly grouped knee joints at a group distance of 10° when evaluating their functional status (25). Therefore, our study assumed a 10° difference between the two groups at 6 weeks and a degree of 125° for patients in the MHI group and 115° for patients in the CON group. Referring to the results of van der List and DiFelice GS (26), the standard deviation was set at 15° for both groups, with a significance level α of 0.05 and 1- β of 0.90. The study was a randomized controlled trial (RCT). The ratio of the number of people in the two groups was 1:1. Calculations were performed according to the formula.


$$N={\left[\frac{\left(z_{{}^{\alpha }\text{​​}╱\text{​}{\text{​}}_{2}\;}+z_{\beta }\right)\sigma }{\delta }\right]}^{2}\left(Q_{1}^{-1}+Q_{2}^{-1}\right)$$

The sample size of each group was calculated to be 48. Considering that the rate of missing secondary follow-up was about 20%, the total sample size was set to 120.

The data in the text and figures are presented as the mean (SD) or median (interquartile range). A modified intention-to-treat analysis was performed that included all patients who were randomized for treatment and attended at least two test sessions. Normality was checked for each variable. The group characteristics of the two groups were compared with a *t-test* when measured using a parametric variable and with the Kruskal–Wallis and chi-square tests when measured using non-parametric and count variables, respectively.

The subjective outcomes were analyzed using a multilevel analysis (SPSS version 24). A random intercept and slope model were used where repeated measurements (level 1) were nested within individual patients who underwent ACL reconstruction (level 2). After that, the following explanatory variables were added to the model: group [MHI and CON (as reference)], time [before surgery (as reference) and 2 and 6 weeks after surgery], and group-by-time interaction. The parameters of the multilevel model were estimated using the maximum-likelihood method. Only models with significantly better log-likelihood values were retained. The secondary outcomes were separately analyzed for the questionnaire and clinical indexes.

Additional multilevel analyses were performed to examine whether sex, BMI, ROM of the non-injured leg, and age affected the recovery after ACL surgery. The multilevel model was identical to the model mentioned above, with the exception that the explanatory variable group was replaced by sex (male or female), BMI (normal, underweight, overweight, or obese), and age (old or young). For age, the patients in the old group were ≥ 30 years old, and those in the young group were < 30 years old. The BMI was < 24 kg/m^2^ in the regular or underweight group, 24 to 28 kg/m^2^ in the overweight group, and ≥ 28 kg/m^2^ (Chinese standard) in the obese group. The target outcomes were the compliance score, VAS score, and the difference in thigh circumference. Cohen's *d* and 95% confidence intervals (CI) were calculated for significant effects. The level of significance (α) was set at *P* < 0.05.

### Ethics

The study protocol was approved by the Ethics Committee of Peking University Third Hospital, authorization number M2019069, and was registered at ClinicalTrials.gov (NCT03890848). In addition, all participants signed an informed consent form. This study conforms to all Consolidated Standards of Reporting Trial guidelines and reports the required information accordingly.

## Results

### Patients

**Figure 3:**
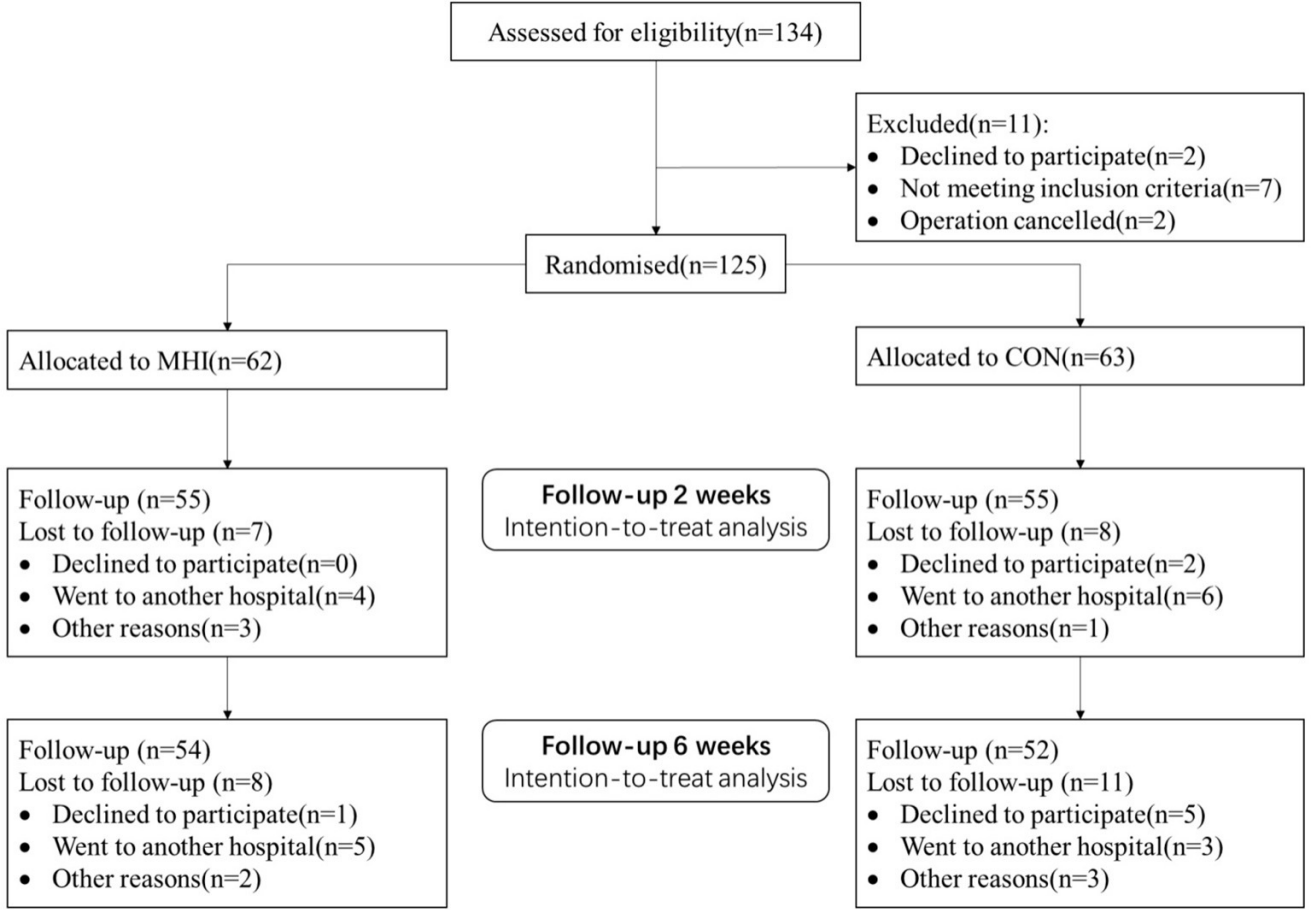
Study participant flowchart (CON, Control group; MHI, mHealth intervention group).

This study was conducted by sequential enrollment. A total of 125 study subjects were included at baseline, 63 in the control group and 62 in the intervention group. The flow of the participants is shown in Figure 3. Table 2 shows the group characteristics of the patients included in the final analysis.

**Table 2:** Mean (SD) baseline characteristics of the participants.

**Characteristics**	**MHI (*n* = 62)**	**CON (*n* = 63)**	***P*-value**
Age (years)	28.9 (7.1)	29.1 (6.8)	0.87
**Gender**			
Male	46	53	
Female	16	10	0.17
Mass (kg)	73.6 (13.6)	77.0 (13.4)	0.16
Height (cm)	173.0 (8.4)	175.3 (7.4)	0.11
BMI (kg/m^2^)	24.5 (3.8)	25.0 (3.4)	0.46
ROM of non-injured leg (°)	140.0 (15.9)	142.3 (11.1)	0.65
Degree of amyotrophy (cm)	1.0 (1.8)	1.0 (2.0)	0.62
Visual analog scale (VAS) scores	2.0 (4.0)	1.0 (3.0)	0.09
IKDC scores	61.0 (11.9)	61.8 (11.9)	0.87
**Combined with meniscus injury**			
Yes	43	43	
No	19	20	0.89
**The injured leg**			
Left	34	39	
Right	28	24	0.42
Time between injury and surgery (days)	206.1 (313.0)	210.3 (318.3)	0.94

CON, Control group; MHI, mHealth intervention group.

Degree of amyotrophy, the circumference difference of 10 cm in the upper margin of patella.

### ROM

At the 2-week follow-up, the qualified rates of ROM in the CON group and MHI group were 40.0 and 47.3%, and the angles were (83.8 ± 16.4)° and (86.6 ± 15.8)°, respectively. The MHI group was slightly higher, but no statistical difference (*P* = 0.361).

At the 6-week follow-up, the qualified rate of ROM in the CON group and the MHI group were 42.6 and 67.3%, respectively, which was statistically different (*P* = 0.008). The angle in the CON group was (118.1 ± 20.5)°, and the angle in the MHI group was (126.6 ± 13.0)°, which was statistically significant (*P* = 0.011).

### The difference in thigh circumference

At the 2-week follow-up, the difference in thigh circumference was 2.0 (1.0, 2.5) cm in the CON group and 1.5 (1.0, 2.5) cm in the MHI group, and the difference between the two groups was not statistically significant (*P* = 0.592). The difference in thigh circumference of the two groups at 2 weeks was higher than before the operation, and there was no statistical difference between the changes in the two groups (*P* = 0.421).

**Figure 4:**
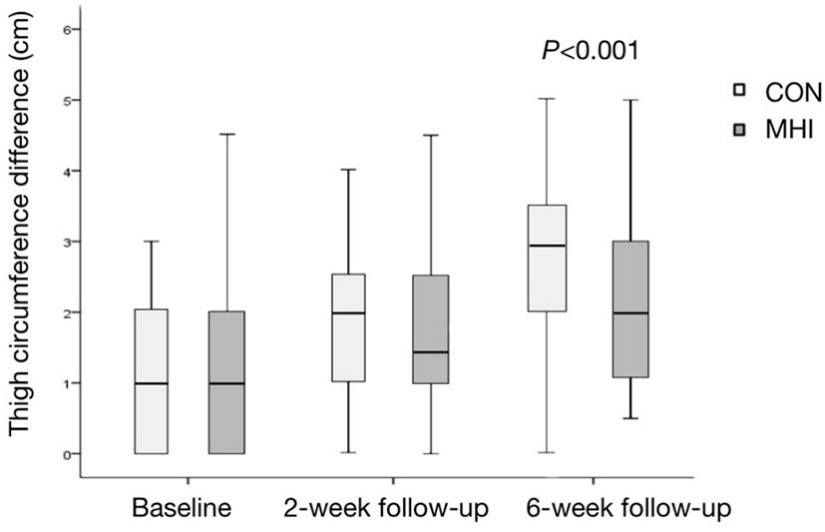
Box plot of thigh circumference difference. At the 6-week follow-up, the difference between the MHI and CON groups was statistically significant (*P* < 0.001). The difference in thigh circumference was more negligible in the MHI group, implying better rehabilitation.

At the 6-week follow-up, the difference in thigh circumference was 3.0 (2.0, 3.5) cm in the CON group and 2.5 (1.0, 3.0) cm in the MHI group, and the difference was statistically significant (*P* < 0.001). The difference in thigh circumference at the 6-week follow-up was still higher than that at the 2-week follow-up, and there was a statistical difference in the change in the difference between the two groups (*P* = 0.038). See Figure 4 for details.

### VAS score

At the 2-week follow-up, the VAS score was 4.0 (3.0, 5.0) in the CON group and 3.0 (3.0, 4.0) in the MHI group. The difference between the two groups was not statistically significant (*P* = 0.435). Compared with the baseline, the VAS scores of the two groups increased at 2 weeks, and there was no statistical difference between the increase in the two groups (*P* = 0.181).

**Figure 5:**
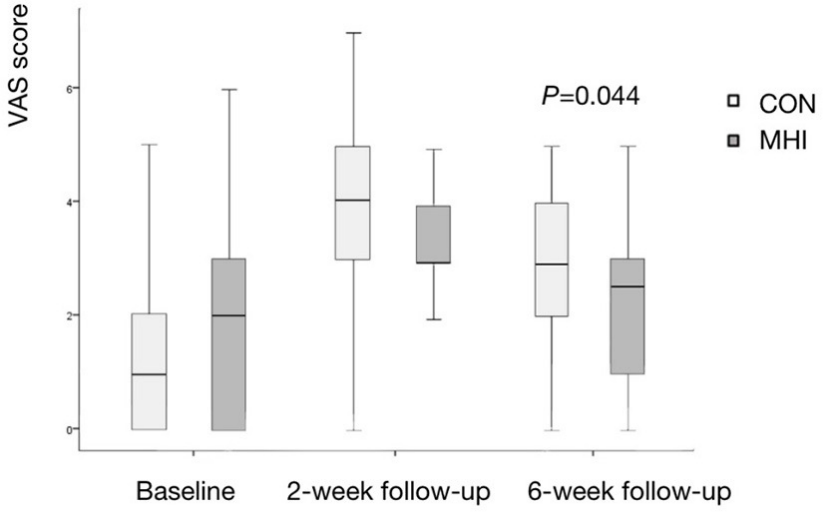
Box plot of VAS score. The difference between the MHI and CON groups was statistically significant at the 6-week follow-up. Less pain in the MHI group was detected, which means better recovery.

At the 6-week follow-up, the VAS score was 3.0 (2.0, 4.0) in the CON group and 2.5 (1.0, 3.0) in the MHI group. The difference was statistically significant (*P* = 0.044). Compared with the 2 weeks, the pain of the two groups was alleviated at 6 weeks, and the change of VAS score between the two groups was not statistically different (*P* = 0.312). See Figure 5 for details.

### IKDC and compliance

There were no statistically significant differences in IKDC scores and compliance scores between the CON group and the MHI group at 2 and 6 weeks of follow-up. A paired *t-test* for 6 weeks and 2 weeks showed that the compliance score of the CON group decreased by 1.4 ± 5.0, and the change in the MHI group was not statistically significant. Through the generalized linear model, after adjusting for age, gender, BMI, education level, and monthly income, it was found that the compliance score of patients in the MHI group was significantly higher than that in the CON group (*P* = 0.047, β = 2.243, 95%CI: 0.026–4.459 ), see Table 3 for details.

**Table 3:** IKDC and compliance scores at 2 and 6 weeks follow-up (mean ± SD).

	**2-week follow-up** ^**a**^			**6-week follow-up** ^**b**^			**2-week follow-up - 6-week follow-up**		
	**CON**	**MHI**	***P-*** **value**	**CON**	**MHI**	***P-*** **value**	**CON**	**MHI**	***P-*** **value**
IKDC	50.9 ± 14.9	54.9 ± 9.8	0.093	56.9 ± 12.5	57.4 ± 12.8	0.813	5.9 ± 13.0	2.5 ± 11.2	0.214
Compliance	37.9 ± 4.5	36.3 ± 5.2	0.087	36.6 ± 3.9	37.3 ± 3.5	0.334	−1.4 ± 5.0^*^	1.0 ± 6.2	0.047

^a^Represents 63 cases in the CON group, 62 cases in the MHI group.

^b^Represents 55 cases in the CON group, 55 cases in the MHI group.

The immobilization was stable for 6 weeks after reconstruction, and no ligament laxity was found. At 6 weeks, the researchers conducted Lachman tests on the patients, and the results were all negative. No significant deep vein thrombosis (DVT) was observed in the patients owing to frequent postoperative ankle pump exercises. All the wounds healed in grade A. Twelve patients (23.1%) in the CON group were reported to have received additional guidance from the rehabilitation institution, which was 18 (33.3%) in the intervention group, showing no statistically significant difference (χ^2^ =1.37, *P* = 0.24). Within 6 weeks, the frequencies of visits in the CON and MHI groups were 13.8 (9.3) and 9.7 (8.6), respectively, without statistically significant differences between the two groups (T = 0.86, *P* = 0.41).

## Discussion

The primary finding of the present study was that for the primary endpoint of objective outcome based on knee joint function, Mhealth-based education had superior outcomes to the traditional paper schedule at 6 weeks.

### Clinical outcomes

#### ROM

Compared with those at 2-week follow-up, the significant and clinically important improvements in all the measures of knee function at 6 weeks that were observed in both the MHI and CON groups are in line with recent literature in patients who underwent ACL reconstruction (17, 27). Studies have shown that ROM is closely related to daily activity needs. Patients can meet the minimum standard of daily activities when they reach ≥90° (ROM) and can easily squat down when the ROM is > 120° (28). The earlier the patients are provided help to achieve greater ROM, the more patients will experience improvements in quality of life and satisfaction (29–31). In the present study, the ROM in the MHI group was slightly higher than that in the CON group at 2-week follow-up after reconstruction, and the values were similar to those in previous studies (32, 33), but the difference was not statistically significant. By 6 weeks, a more remarkable improvement in ROM was observed in the MHI group than in the CON group, similar to the results of a previous study (31). This is mainly related to the following two factors: First, the content of the patient's training 1 week after reconstruction was the basic training, and the content was increased after that. The patients in the MHI group had access to more engaging and varied instruction, such as pictures, videos and audio. Second, as time passed, the patients' attention to rehabilitation training showed a downward trend, and psychological barriers gradually increased, as demonstrated in previous studies (34, 35). At this time, timely mHealth-education and prescription could motivate patients to maintain relatively high compliance to achieve better rehabilitation effects.

#### QF

The degree of atrophy of the quadriceps femoris (QF) muscle is recognized as one of the critical factors of poor knee function despite successful ACL reconstruction (36). The QF muscle activity is reduced due to problems such as limited knee flexion after ACL reconstruction. Meanwhile, skeletal muscle and ligament injuries cause a shortage of oxygen supply and damage to the adjacent capillaries, which leads to QF atrophy (37, 38). Previous studies showed an interaction between joint pain and QF atrophy, which might worsen with each other (39, 40). In the present study, the patients who underwent MHI received frequent training reminders, which led to better performance in reducing QF atrophy. The reduction in QF atrophy with greater pain reduction may have contributed to a greater improvement in ROM (41). No related complications were found between the two groups, and no statistically significant differences in the number and frequency of extra visits to medical institutions were observed.

#### Flexion pain

Flexion pain after surgery is one of the essential reasons for patient dissatisfaction and increased incidence of secondary surgery, and standardized training plays an irreplaceable role in pain relief (42). Normative training can improve the medial femoral track and correct the abnormal patellofemoral joint, thus reducing patellofemoral pain syndrome (43). Another way to reduce postoperative flexion pain is to use an intensive icing regimen (44, 45). The two important aspects of the MHI were to offer normative training guidance and a timely icing regimen reminder, which contributed to the more significant reductions in flexion pain in the MHI group, ensuring that patients could normally train as expected. In addition, detailed surgical introduction, rehabilitation case sharing, and training guidance can enhance patients' perceptions of the training intensity, thus reducing patients' fear avoidance and ensuring a good target-reach rate, similar to a previous study (46).

### Subjective functional outcomes

At 2 and 6 weeks after surgery, the IKDC score in the MHI group was slightly higher than in the CON group, but the difference was not statistically significant. The scores in both groups improved, but the difference between the two groups decreased. In addition, all the patients who underwent ACL reconstruction at Peking University Hospital received detailed explanations on how to deal with pain at the time of discharge from the hospital. The pain levels of the patients were kept low. At the same time, in this study, the process of isolated ACL reconstruction was unified and standardized, and the wound healing of the patients was good, without complications during the 6-week follow-up, so no significant difference was found in the activities that could be performed. These results might explain why the two groups had no statistically significant difference in IKDC scores.

Regarding compliance, no significant difference was found in the scores between the two groups, which might indicate that within 6 weeks after the operation, all the patients could undergo rehabilitation training on time, following the rehabilitation plan. However, the understanding and standardization of specific actions showed differences, which led to differences in the clinical index.

## Limitations

This study focused on early knee function in patients who underwent ACL reconstruction, and its long-term impact remains to be further investigated. Owing to the late opening of the browsing record collection function, the Mhealth platform's usage record is not yet complete, so further evaluation of the patients' usage will be considered in the next step. Mhealth intervention requires patients to learn to use smartphones and corresponding software, so promotion has certain limitations. Considering this factor, in the later stage, you can consider adding an education column for caregivers in the mHealth platform and implementing interventions by intervening with caregivers.

## Conclusion

Standardized rehabilitation exercises after surgery improved knee joint function and reduced muscle atrophy and flexion pain. Mhealth-based education further promoted the improvement of clinical indicators but did not significantly improve the subjective results after ACL reconstruction.

## Data availability statement

The original contributions presented in the study are included in the article/supplementary material, further inquiries can be directed to the corresponding authors.

## Ethics statement

The studies involving human participants were reviewed and approved by Peking University Third Hospital Medical Science Research Ethics Committee. The patients/participants provided their written informed consent to participate in this study.

## Author contributions

YG and DL were involved in data analysis and data interpretation. X-yS designed the study. All authors were involved in data collection and analysis as well as the write-up of the final manuscript.
